# The Ingenious Structure of Central Rotor Apparatus in V_o_V_1_; Key for Both Complex Disassembly and Energy Coupling between V_1_ and V_o_

**DOI:** 10.1371/journal.pone.0119602

**Published:** 2015-03-10

**Authors:** Atsuko Nakanishi, Jun-ichi Kishikawa, Masatada Tamakoshi, Ken Yokoyama

**Affiliations:** 1 Department of Molecular Biosciences, Kyoto Sangyo University, Motoyama Kamigamo, Kita-ku, Kyoto, Japan; 2 Department of Molecular Biology, Tokyo University of Pharmacy and Life Science, Horinouchi, Hachioji, Tokyo, Japan; University of Cambridge, UNITED KINGDOM

## Abstract

Vacuolar type rotary H^+^-ATPases (V_o_V_1_) couple ATP synthesis/hydrolysis by V_1_ with proton translocation by V_o_ via rotation of a central rotor apparatus composed of the V_1_-DF rotor shaft, a socket-like V_o_-C (eukaryotic V_o_-d) and the hydrophobic rotor ring. Reconstitution experiments using subcomplexes revealed a weak binding affinity of V_1_-DF to V_o_-C despite the fact that torque needs to be transmitted between V_1_-DF and V_o_-C for the tight energy coupling between V_1_ and V_o_. Mutation of a short helix at the tip of V_1_-DF caused intramolecular uncoupling of V_o_V_1_, suggesting that proper fitting of the short helix of V_1_-D into the socket of V_o_-C is required for tight energy coupling between V_1_ and V_o_. To account for the apparently contradictory properties of the interaction between V_1_-DF and V_o_-C (weak binding affinity but strict requirement for torque transmission), we propose a model in which the relationship between V_1_-DF and V_o_-C corresponds to that between a slotted screwdriver and a head of slotted screw. This model is consistent with our previous result in which the central rotor apparatus is not the major factor for the association of V_1_ with V_o_ (Kishikawa and Yokoyama, J Biol Chem. 2012 24597-24603).

## Introduction

The Vacuole-type ATPases (V_o_V_1_) are found in many organisms and are involved in a variety of physiological processes [1–3]. V-ATPases in eukaryotic cells (eukaryotic V_o_V_1_) translocate protons across the membrane consuming ATP. Most prokaryotic V_o_V_1_ (also referred to as A-ATPase or A_o_A_1_ [1, 4]) produce ATP using the energy stored in a transmembrane electrochemical proton gradient [3, 5], while the V_o_V_1_ of some anaerobic bacteria, such as *Enterococcus hirae*, function as a sodium pump [6].

**Fig 1:**
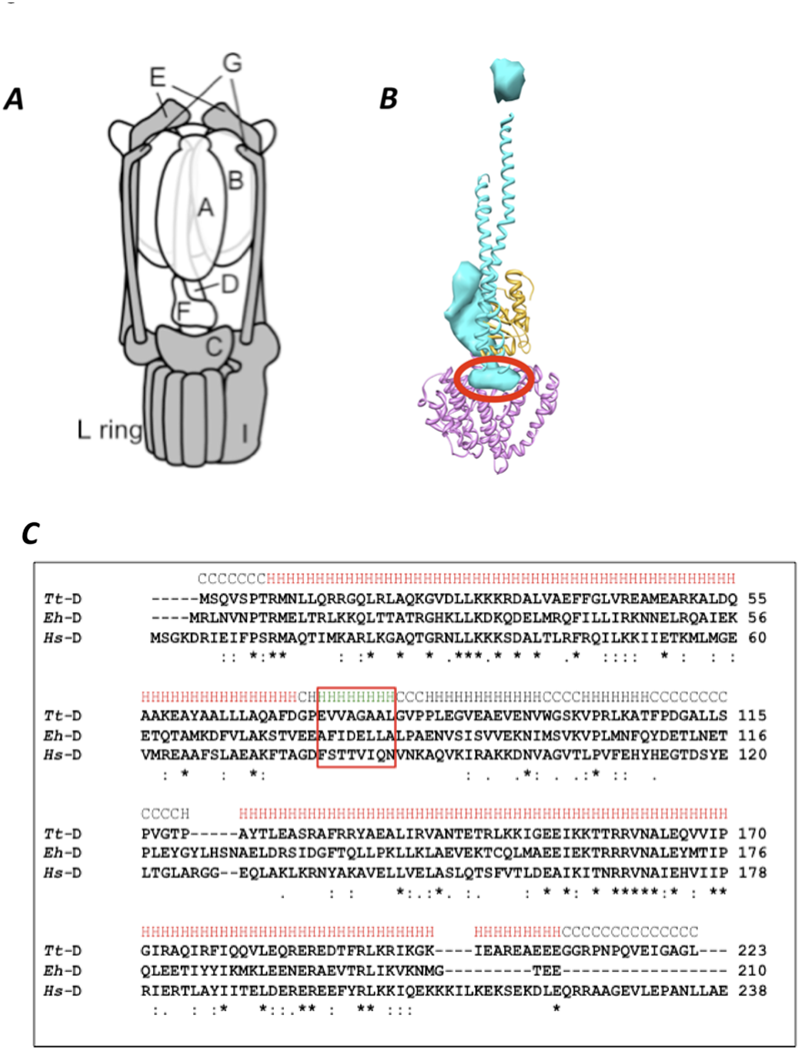
V_o_V_1_ and the short helix of V_1_-D subunit. A, Schematic representation of *T*. *thermophirus* V_o_V_1_ [16]. Subunits in V_o_ and V_1_ are shown in *gray* and *white*, respectively. B, The structure of the central rotor apparatus of V_o_V_1_ obtained by EM density map (PDBID; 3J0J) with the short helix in V_1_-D subunit circled in red. The V_1_-D, V_1_-F and V_o_-C subunits are represented in blue, yellow, and pink, respectively. C, Sequence alignment of V_1_-D subunit of *T*. *thermophilus (Tt)*, *E*. *hirae (Eh)* and *H*. *sapiens (Hs)*. Identical amino acid residues are represented by asterisks. The sequences of the short helix of the V_1_-D subunit are surrounded by the red box.

The V_o_V_1_ and F_o_F_1_ ATPases/synthases are evolutionarily related and share a rotary mechanism to perform their specific functions [3, 7–9]. The basic structures of the ATPases/synthases are conserved among species [1–3]. The soluble, cytoplasmic portion of F_o_F_1_ and V_o_V_1_ (called F_1_ and V_1_, respectively), responsible for ATP hydrolysis/synthesis, is connected via the central rotor stalk and the peripheral stator stalk to the transmembrane portion (F_o_ and V_o_) that houses the ion transporting pathway [1–3]. In *Thermus thermophilus* V_o_V_1_, the V_1_ portion is composed of a hexameric A_3_B_3_ cylinder and a central shaft comprised of the D and F subunits [3, 10, 11]. The V_o_ portion is composed of 5 different subunits with a stoichiometry of C_1_E_2_G_2_I_1_L_12_ (see Fig. 1A). In F_o_F_1_, a γ -subunit (equivalent to subunits D and F of V_o_V_1_) binds directly to the rotor ring [12]. In contrast, at the boundary surface of V_o_V_1_, V_o_-C forms a socket-like structure which accommodates the V_1_-DF central shaft [13], indicating that V_1_-DF does not contact the rotor ring directly. Thus, the boundary surface of V_o_V_1_ is significantly different from that in F_o_F_1_. V_o_V_1_ also has a more complex peripheral stalk structure than F_o_F_1_. The stator structure of F_o_F_1_ consists of a single peripheral stalk, while V_1_ is connected with V_o_ by two or three peripheral stalks [4, 14, 15].

Recent reconstitution studies of *T*. *thermophilus* V_o_V_1_ have demonstrated that the A_3_B_3_ domain is tightly associated with the two EG peripheral stalks of V_o_, even in the absence of the central shaft subunits [16]. In other words, the peripheral stalks are the major factor mediating association of V_1_ and V_o_, consistent with the unique boundary surface between V_1_-DF and V_o_-C in V_o_V_1_. This arrangement is highly relevant for the detachment of V_1_-DF from V_o_-C [13, 16]. However torque needs to be transmitted between V_1_-DF and V_o_-C for tight energy coupling between V_1_ and V_o_ [16]. Thus a sticky interaction which also allows detachment of V_1_-DF from V_o_-C is required. How the protein maintains these two somewhat contradictory properties has yet to be investigated.

Lau *et al*. reported a sub-nanometer resolution structure of *T*. *thermophilus* V_o_V_1_ by single particle cryo-electron microscopy [11] showing that the rod like structure of V_1_-DF is positioned in the cavity of V_o_-C. This suggests that the rod like structure might play an important role in binding of V_1_-DF and V_o_-C. A crystal structure of V_1_-DF isolated from *E*. *hirae* V_o_V_1_ suggested that the rod like structure might be a short helix at the tip of the V_1_-DF (Fig. 1B, [17]).

In this study, reconstitution and fluorescence resonance energy transfer (FRET) analysis of V_o_V_1_ subcomplexes reveal that the binding affinity of V_1_-DF with V_o_-C subunit is weak. Further investigations indicated that the short helix of the V_1_-DF subunit has important roles in both reconstitution of V_o_V_1_ and torque transmission. We propose a structural model accounting for both the detachable and sticky nature of the interaction between V_1_-DF and V_o_-C.

## Materials and Methods

### Proteins isolation of V_o_ and CL_12_

Wild-type or mutant V_o_V_1_ (C-T105C/C-C268S/C-C323S) from *T*. *thermophilus* strains incorporating a His_3_ tag on the C terminus of subunit L were generated by the integration vector system [18]. Culture of the modified *T*. *thermophilus* strains, membrane preparation, solubilization of His-tagged V_o_V_1_ and purification of V_o_V_1_, V_o_ and CL_12_ were carried out as described previously [19]. The mutated V_o_ and CL_12_ (C-T105C/C-C268S/C-C323S) were used for the FRET experiments.

### Preparation of V_1_ (A_3_B_3_DF) and V_1_-DF

*Escherichia coli* strain BL21-CodonPlus-RP (Stratagene) was used for expression of V_1_ (A_3_B_3_DF) and V_1_-DF. These recombinant subcomplexes were isolated as described previously [20, 21]. The expression plasmids for V_1_ containing DF from *H*. *sapiens* or *E*. *hirae* were constructed by the same method as described in ref. [21]. The genes encoding the D and F subunits were amplified from human cDNA and pCemtp18 [22], containing the complete *E*. *hirae* V_o_V_1_ (ntp) operon. The D and F genes of the *T*. *thermophilus* V_1_ expression plasmid were replaced with the amplified genes. The expression plasmids for mutated V_1_ lacking the short helix or containing the swapped short helix of *E*. *hirae* were constructed by PCR mutagenesis. To introduce the swapped short helix of *E*. *hirae*, complementary oligonucleotide primers containing the sequences encoding the 8 amino acids of this region were used to amplify the gene fragment. The fragment was then digested with appropriate restriction enzymes, and inserted into the corresponding region of the *T*. *thermophilus* V_1_ expression plasmid [20]. The mutant V_1_ (A-His_8_/ΔCys, A-C255A/A-S232A/A-T235S, F-S54C) and mutant DF (F- His_6_, S54C) were used for either reconstitution or FRET experiments [16].

### Reconstitution of V_o_V_1_

The purity of each subcomplex was confirmed by SDS-PAGE. V_1_ or V_1_-DF (each 1 mg/ml) in MOPDM buffer (50 mM MOPS (pH 7.0), 150 mM NaCl, 0.03% n-dodecyl-β-D-maltoside) was mixed with 1 mg/ml V_o_ or CL_12_ at an equal volume ratio. The mixtures were incubated for 1 h at 25°C and then applied onto the Superdex HR-200 column equilibrated with the same buffer. The reconstituted V_o_V_1_ were collected and used for further analysis immediately.

### FRET analysis

FRET analysis was carried out as described previously [16]. The purified V_1_ (A-His_8_/ΔCys, A- S232A/A-T235S, F-S54C) or DF (F-His_6_, S54C) was immediately labeled with an excess amount of Cy3-maleimide (GE healthcare, used as a donor molecule) in MOPDM buffer. Following a 60 min incubation at 25°C, proteins were separated from unbound reagent with a PD-10 column (GE Healthcare). The mutated V_o_ (C-T105C/C-C268S/C-C323S) was labeled with Cy5-maleimide (GE Healthcare, used as an acceptor molecule) by the same method described above. The specific labeling of subunit F in V_1_ or DF, and subunit C in V_o_ or CL_12_ was checked by measurement of subunit fluorescence. FRET, as a result of reconstitution of V_o_V_1_, was monitored with a fluorimeter using an excitation wavelength of 532 nm and an emission wavelength of 570 nm (FP-6200, JASCO). A cuvette was filled with 1.2 ml of MOPDM buffer containing 2 nM labeled V_1_ or DF and incubated at 25°C until the fluorescence intensity reached a constant level. For measurement of binding kinetics, 8.0 μl of labeled V_o_ or CL_12_ was added into the cuvette at a final concentration of 10 nM.

### Mesurements of ATP synthesis of the V_o_V_1_

The reconstituted complexes were incorporated into liposomes using a freeze-thaw method [23]. Acidification of the proteoliposomes and measurement of ATP synthesis were carried out at 25°C. To acidify the interior of proteoliposomes, 30 μl of the proteoliposome solution was mixed with 15 μl of acidification buffer (300 mM MES pH4.7) and then incubated for 5 min at 25°C. The ATP was measured as the increase of intensity of luminescence in a Luminescencer-PSN (ATTO). The ATP synthesis reaction was initiated by injection of 30 μl of the acidified proteoliposomes into 0.5 ml of base buffer containing 100 mM Tricin-sodium (pH 8.5), 2.5 mM MgSO_4_, 10 mM phosphate, 2.2 mg of luciferin/luciferase compound, 0.5 mM ADP, 36 nM valinomycin, and 100 mM KCl. For calibration, ATP was injected into the base buffer.

### Measurements of proton channel and proton pump activity by the V_o_V_1_

Crude soybean L-α-Phosphatidylcholine (Sigma) was washed with 20 mM Tricine-sodium (pH 8.5), 2.5 mM MgSO_4_ to remove K^+^ as described [24]. K^+^-loaded proteoliposomes containing enzyme were prepared as follows; aliquots containing reconstituted complex were diluted to 0.5 mg/ml in 20 mM Tricine, 2.5 mM MgCl_2_ 20 mM MES pH8.0, 4% *n*-Octyl-β-D-glucoside (Sigma), the washed 20 mg/ml lipid and 150 mM KCl for the measurement of proton pump or 500mM KCl for the measurement of proton channel activity. Bio-beads SM-2 (Bio-Rad) were added to remove the detergent and incubated for 2 h at 25°C. Resultant proteoliposomes were centrifuged and subjected to the proton pump and proton channel analysis.

The acidification of liposomes was measured by the quenching of 9-amino-6-chloro-2-methoxyacridine, (ACMA) (Sigma) fluorescence [25]. Aliquots containing 5 μg of protein were suspended in 1.2 ml of 20 mM Tricine, 2.5 mM MgCl_2_, 40 mM MES pH8.0, 500 mM NaCl for the measurement of proton channel or 110 mM NaCl /40 mM KCl for the measurement of proton pump activity, in the presence of 15 ng of ACMA. The time course of fluorescence quenching was monitored using a fluorimeter (FP-6200, JASCO).

### Other assays

Protein concentrations of V_1_ were determined from UV absorbance calibrated by quantitative amino acid analysis; 1 mg/ml gives an optical density of 0.88 at 280 nm. Protein concentrations of V_o_ and V_o_V_1_ were determined by BCA protein assay, with BSA used as the protein standard. ATPase activity was measured at 25°C with an enzyme-coupled ATP regenerating system [5]. Polyacrylamide gel electrophoresis in the presence of SDS or AES was carried out as described previously [5]. The proteins were stained with Coomassie Brilliant Blue.

## Results

### Weak interaction at the boundary surface between V_1_-DF and CL_12_

**Fig 2:**
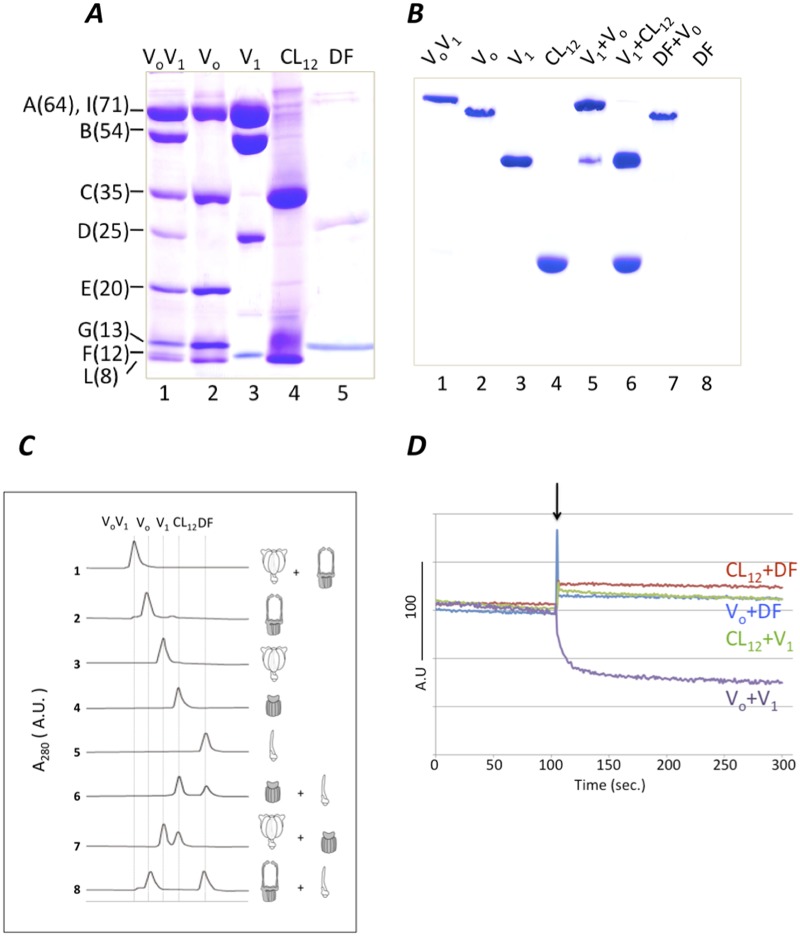
Analysis of reconstitution of complexes and subcomplexes. A, 15% SDS-PAGE analysis. *Lane* 1, V_o_V_1_; *lane* 2, V_o_; *lane* 3 V_1_; *lane* 4, CL_12_; *lane* 5, DF. Molecular weights of each subunit are indicated in parentheses. B, 6% AES-PAGE. The mixtures containing the subcomplexes were incubated for 1h at 25°C respectively, prior to analysis. *Lane* 1, V_o_V_1_; *lane* 2, V_o_; *lane* 3, V_1_; *lane* 4, CL_12_; *lane 5*, V_o_ and V_1_; *lane 6*, V_1_ and CL_12_; *lane 7*, DF and V_o_, *lane 8*; DF. The band of DF complex was not detected on AES-PAGE gel. C, Gel permeation analysis of complexes and subcomplexes. The mixtures containing subcomplexes indicated by the scheme were incubated for 1h at 25°C respectively, followed by analysis. The molecular weights of each complex are, V_o_V_1_ (659kDa), V_o_ (268kDa), V_1_ (391 kDa), CL_12_ (131 kDa) and V_1_-DF (37 kDa). D, FRET analysis of reconstituted complexes and subcomplexes. Fluorescence of 3 nM V_1-Cy3_ (purple and green lines) or 3 nM V_1_-DF_-Cy3_ (blue and red lines) was recorded (excitation at 532nm) before and after addition of V_o-Cy5_ or CL_12-Cy5_. The V_o-Cy5_ or CL_12-Cy5_ was added at the time indicated by the arrow.

Reconstitution of A_3_B_3_ and V_o_ has indicated that the interaction between A_3_B_3_ and two EG peripheral stalks is rigid [16]. However the precise nature of the interaction between V_1_-DF and V_o_-C has not been experimentally characterized. In order to examine the interaction the isolated V_1_-DF subunits or subcomplexes from V_1_ with V_o_ or CL_12_, were mixed with V_o_ or CL_12_. The purity and subunit stoichiometry of all subcomplexes were confirmed by both SDS-PAGE and AES-PAGE (Fig. 2A and B). The band corresponding to V_1_-DF was not detected on AES-PAGE gel. Reconstituted V_o_V_1_ was identified as a single band on AES-PAGE when V_1_ was incubated with V_o_ at a molar ratio of 1:1 (Fig. 2B, lane 5). However attempts to reconstitute V_1_ with CL_12_ were unsuccessful as assessed by both AES-PAGE and gel permeation analysis (Fig. 2B, lane 6 and Fig. 2C, line 7). The same was also true for V_1_-DF and CL_12_ or V_o_ (Fig. 2B, lane 7 and Fig. 2C, line 6 and 8). Together, these results strongly suggest that the interaction of the boundary surface between V_1_-DF and V_o_-C in the complex is insufficient for reconstitution of a stable complex.

Further analysis of the interaction at the boundary surface between V_1_-DF and V_o_-C was carried out by fluorescent resonance of energy transfer (FRET), a powerful method for detecting protein-protein interaction/association [16]. For FRET analysis, each component was labeled with Cy3 or Cy5 (fluorescent dyes as described in ref. [16]). A mutated V_1_-DF or V_1_ incorporating a single cysteine residue (F/S54C) was labeled with Cy3 as a donor, while a mutated V_o_ or CL_12_ (C/T105C) was labeled with Cy5 as an acceptor. Reconstitutions were carried out in a cuvette containing 1.2 ml of the 2 nM V_1_ or V_1_-DF labeled with Cy3. The reconstitution efficiency was evaluated by the decrease in donor emission (Fluorescence at 570 nm, ref. [16]). As shown in Fig. 2D, the fluorescence of V_1_ decreased sharply upon addition of V_o_ into the cuvette, indicating reconstitution of V_1_ and V_o_ complex (purple line). In contrast, addition of 8.0 μl of 1.5 mM of CL_12_ or V_o_ into a cuvette containing V_1_-DF exhibited no decrease in fluorescence at 570 nm (red or blue lines). Furthermore the addition of 8.0 μl of 1.5 mM of CL_12_ into a cuvette containing V_1_ showed no decrease in fluorescence (green line). These results clearly indicate low binding affinity between V_1_-DF and V_o_-C, consistent with the findings from the reconstitution experiments [16].

### Effect of exogenous V_1_-DF on reconstitution of V_1_ and V_o_

**Fig 3:**
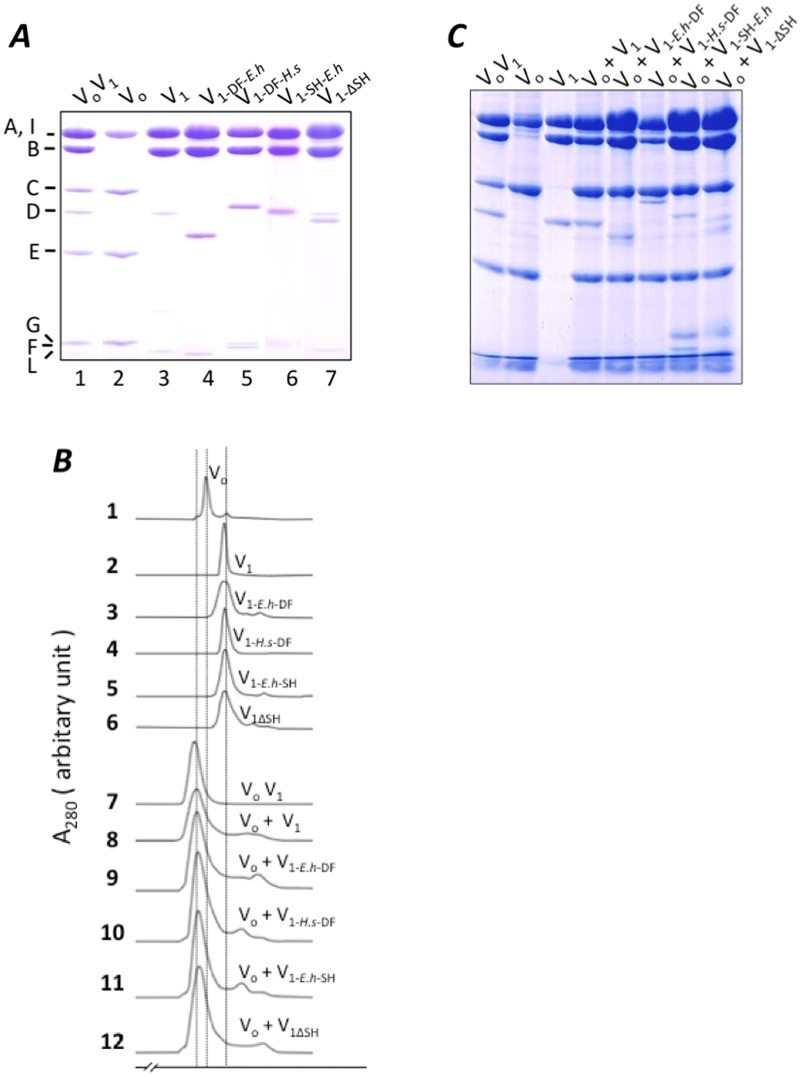
Analysis of reconstituted V_1_ with V_o_. Isolated V_1_ and V_o_ were analyzed by SDS-PAGE (A). *Lane* 1, V_o_V_1_; *lane* 2, V_o_; *lane* 3, V_1_; *lane* 4, chimeric V_1_ containing *E*. *hirae* V_1_-DF (V_1-*Eh*-DF_); *lane* 5, chimeric V_1_ containing *H*. *sapiens* V_1_-DF (V_1*Hs*-DF_); *lane* 6, mutated V_1_ containing the exogenous short helix of *E*. *hirae* (V_1SH-*Eh*_); *lane* 7, the mutated V_1_ lacking the short helix (V_1ΔSH_). Analysis of reconstituted mutated V_1_ and V_o_ by gel permeation (B). The mixtures containing the mutated V_1_ and V_o_ were incubated for 1 h at 25°C, prior to analysis. Subunit stoichiometry of the fraction containing each mutated V_o_V_1_ was confirmed by SDS-PAGE analysis (C).

The amino acid sequence of V_1_-DF is not highly conserved among species (Fig. 1C). To investigate the effect of theses differences on reconstitution of V_1_ and V_o_, expression constructs of V_1_ containing V_1_-DF from *Homo sapiens* (V_1-DF-*H*.*s*_) or *E*. *hirae* (V_1-DF-*E*.*h*_) were generated. These chimeric V_1_ were purified and subjected to ATPase analysis as described in Materials and Methods. Subunit stoichiometry of each chimeric V_1_ was confirmed by SDS-PAGE analysis (Fig. 3A). These chimeric V_1_ were ATPase active (Table 1, S1 Fig.), indicating that the exogenous V_1_-DF functions as a rotor in A_3_B_3_ of *T*. *thermophilus*. As shown in Table 1, the presence of the exogenous DF shaft from *H*. *sapiens* markedly enhanced the ATPase activity of V_1_. The enhanced ATPase activity of chimeric V_1_ is likely due to primary sequence difference between the DF of *T*. *thermophilus* and that of *H*. *sapiens*. We will discuss the effect of the *H*. *sapiens* DF on the activity of V_1_ fully elsewhere.

**Table 1:** Kinetics parameters of the mutated V_1_ for ATPase activity.

	V_1_	V_1-*E*.*h*-DF_	V_1-*H*.*s*-DF_	V_1-SH-*E*.*h*_	V_1ΔSH_
*K*_m_ [mM]	0.23 ± 0.05	0.76 ± 0.04	0.48 ± 0.04	0.22 ± 0.05	0.24 ± 0.06
*V*_max_ [s^-1^]	30.6 ± 1.8	38.0 ± 0.80	132 ± 3.0	27.5 ± 1.5	21.8 ± 1.4

*K*_m_ and *V*_max_ values represent means ± SD (n = 3).

Each chimeric V_1_ was mixed with an excess amount of V_o_ from *T*. *thermophilus* and incubated for 1 hour. Analysis by gel permeation analysis revealed peaks corresponding to reconstituted V_o_V_1_ containing the exogenous V_1_-DF (Fig. 3B, lines 9 and 10). Subunit stoichiometry of the fraction containing each chimeric V_o_V_1_ was confirmed by SDS-PAGE analysis (Fig. 3C). These results indicate that the chimeric V_1_ incorporating the exogenous V_1_-DF can assemble with V_o_.

### Role of the short helix of V_1_-D subunit in interaction of V_1_ and V_o_

The EM density structure of intact V_o_V_1_ of *T*. *thermophilus* and the crystal structure of V_1_-DF suggested that an α-helix of V_1_-D (a.a. 73–80) is key for interaction between V_1_-DF and V_o_-C ([11,17] and Fig. 1B and C). This helix is referred to as the short helix hereafter. To investigate the role of the short helix in reconstitution of V_1_ and V_o_, V_1-SH-*E*.*h*_ containing the exogenous short helix of *E*. *hirae* (A^74^FIDELLA^81^, Fig. 1C), and V_1ΔSH_ lacking the short helix of the V_1_-DF were constructed. The subunit stoichiometry and purity of the mutated V_1_ constructs were confirmed by SDS-PAGE analysis (Fig. 3A). Reconstitution of V_o_ and V_1-SH-*E*.*h*_ or V_1ΔSH_ was confirmed by gel permeation and FRET analysis (Fig. 3B, line 11, 12). Subunit stoichiometry of the fraction containing each mutated V_o_V_1_ was confirmed by SDS-PAGE analysis (Fig. 3C). These findings indicate that the short helix is not essential for reconstitution of V_1_ with V_o_.

### Role of the short helix of V_1_-D subunit in energy coupling between V_1_ and V_o_

**Fig 4:**
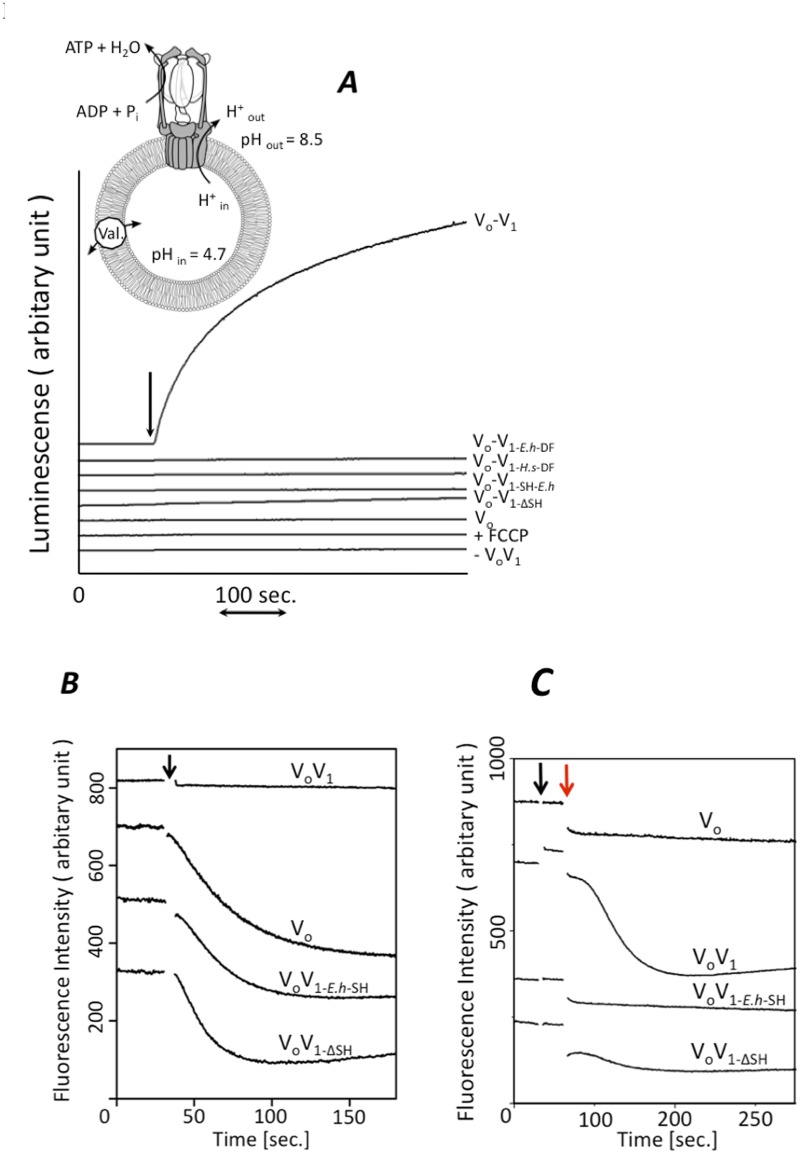
Function of the short helix of V_1_-D subunit for energy coupling. A, Analysis of ATP synthesis by the reconstituted complexes. *inset*; schematic model of the experimental system [16]. The reconstituted V_o_V_1_ was incorporated into liposomes, then energized by an acid base transition procedure described in the Materials and Methods. Each line shows the raw data for ATP synthesis by each reconstituted complex at a Δ pH of 3.8. The reactions were initiated by addition of acidified proteoliposomes into the base buffer, as indicated by the arrow. Final concentrations were 2 μg/ml of V_o_V_1_ and 1 mM ADP. The synthesized ATP was monitored by the luciferin-luciferase assay [18]. Analysis of proton channel (B) and proton pump activity (C) coupled with ATP hydrolysis by proteoliposomes. K^+^-loaded proteoliposomes containing the reconstituted complexes were prepared as described in the Materials and Methods. Proton influx was initiated by addition of 20 ng of valinomycin at the time indicated by the black arrow (B) and proton pump was initiated by addition of ATP-Mg at finally 1mM followed by the addition of valinomycin at the time indicated by the red arrow (C). The ACMA fluorescence emission (480nm) was recorded at 25°C.

To further investigate the role of the short helix of the V_1_-D subunit in energy coupling between V_1_ and V_o_, ATP synthesis activity of the reconstituted V_o_V_1_ were assessed. The reconstituted V_o_V_1_ constructs were purified by gel permeation chromatography and reconstituted into liposomes by freeze-thaw methods. Proton motive force was generated across the membranes of the reconstituted liposomes by acid-base transition (ref. [23], see *inset* in Fig. 4A). As shown in Fig. 4A, wild type reconstituted V_o_V_1_ showed continuous ATP synthesis. In contrast, the reconstituted V_o_V_1ΔSH_ showed no ATP synthesis activity, indicating that lack of the short helix in V_o_V_1_ causes an intra molecular uncoupling between V_1_ and V_o_. In addition, the reconstituted V_o_V_1_ including the exogenous V_1_-DF (V_o_V_1-DF-*H*.*s*_, V_o_V_1-DF-*E*.*h*_) or the short helix (V_o_V_1-SH-*E*.*h*_) showed no ATP synthesis activity.

Next, proton channel and proton pump activity of the reconstituted V_o_V_1_ were measured to investigate the energy coupling efficiency between the mutated V_1_ and V_o_ in the complexes. To facilitate ATP hydrolysis activity measurements, a mutated V_1_ incorporating the TSSA substitutions (A-S232A/A-T235S) to overcome ADP inhibition was used [5]. The wild-type V_o_V_1_ did not show proton channel activity but did show proton pump activity (Fig. 4B, C). These results indicate that the wild-type V_o_V_1_ is tightly coupled. In contrast, mutated V_o_V_1_ including the exogenous short helix or lacking the short helix did not show proton pump activity (Fig. 4C). In contrast, the mutated V_o_V_1_s had proton pump activity almost identical to the proton channel activity of V_o_ (Fig. 4B), indicating that the mutated V_o_V_1_s were completely uncoupled. These results strongly suggest that a proper match between the short helix in V_1_-D subunit and V_o_-C subunit is essential for tight energy coupling between V_1_ and V_o_.

## Discussion

In this study, we have directly demonstrated a low binding affinity between V_1_-DF and V_o_-C by both FRET and reconstitution experiments (Fig. 2). This is consistent with our previous result indicating that the two EG peripheral stalks are the major mediators of association of V_1_ with V_o_ [16]. This low binding affinity between V_1_-DF and V_o_-C is relevant to reversible dissociation/association of V_1_ from V_o_ in eukaryotic V_o_V_1_ [26, 27]. However such low binding affinity is unfavorable for energy coupling between V_1_ and V_o_; for ATP synthesis (ΔG = ~55 kJ/mol, [16]), the torque generated in the V_o_ rotor ring needs to be transmitted to V_1_-DF via V_o_-C. Thus, an ingenious structure, that is both detachable and sticky, is required at the boundary surface between V_1_-DF and V_o_-C. The EM density map of *T*. *thermophilus* V_o_V_1_ [11] provided a clue to unraveling the molecular basis of these seemingly contradictory properties. The short helix of V_1_-DF apparently lies in the cavity of V_o_-C in V_o_, suggesting that it may play an important role in association of V_1_-DF and V_o_-C.

Here, we have investigated the role of the short helix in V_1_-D subunit on V_o_V_1_ assembly and energy coupling between V_1_ and V_o_. Not only the chimeric V_1_ containing the exogenous V_1_-DF and the short helix, but also V_1_ lacking the short helix could reconstitute the complex with V_o_ (Fig. 3), indicating that the short helix is not a key factor for V_o_V_1_ complex formation. However, these mutant V_o_V_1_s showed neither ATP synthesis or proton pump activities (Fig. 4). As shown in Fig. 1C, amino acid residues in the short helix of V_1_-D are not highly conserved among species (*T*. *thermophilus*, *E*. *hirae*, *H*. *sapiens*). The different surface shape of the exogenous short helix compared to the endogenous one likely generates repulsion between V_1_-DF and V_o_-C. The intact fitting of the short helix into the recess of V_o_-C appears to be required for tight energy coupling between V_1_ and V_o_.

**Fig 5:**
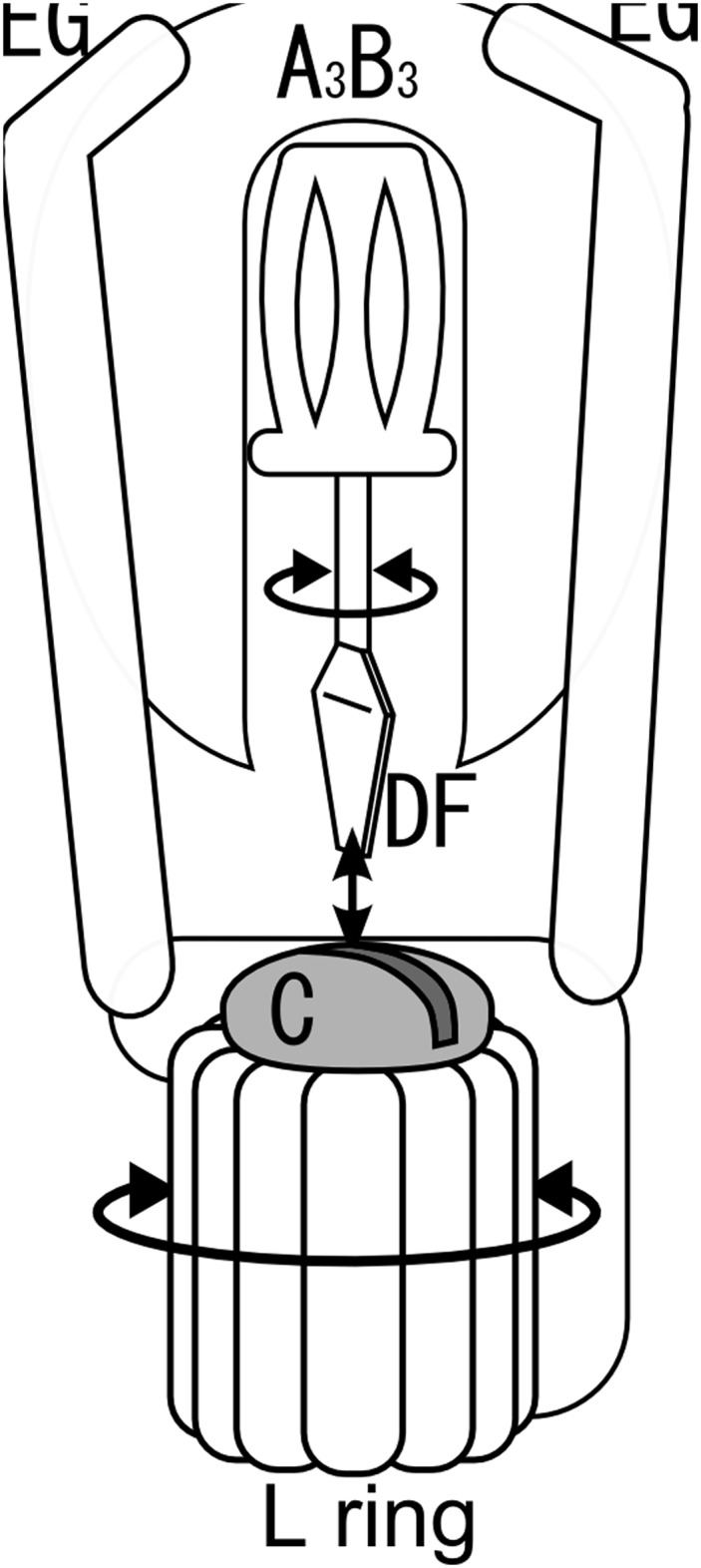
A schematic diagram for a slotted screwdriver and a head of slotted screw in V_o_V_1_ [16].

Here we propose a model to account for a detachable V_1_-DF/V_o_-C boundary surface, which can transmit torque. In this model, the relationship between V_1_-DF and V_o_-C is analogous to that between a slotted screwdriver and a head of slotted screw (Fig. 5). In the V_o_V_1_, two EG peripheral stalks push the short helix of V_1_-D into the socket of V_o_-C. Thus, the short helix of V_1_-D binds into the socket of V_o_-C, forming a sufficiently close interaction for transmission of torque from the rotating V_1_-DF to V_o_-C. The rigid interaction between the V_1_-DF and V_o_-C would be abolished by a loss of interaction between the two EG peripheral stalks and A_3_B_3_, consistent with the results of reconstitution experiments (Fig. 2) and EM analysis of sequential disassembly of the V_o_V_1_ [28]. F_o_F_1_ does not contain a counterpart of V_o_-C so that the F_1_ shaft composed of the γ subunit directly attaches to the c_6–10_ rotor ring [12]. Thus, the F_1_-c_6–10_ stator-less complex is easily isolated from F_o_F_1_.

## Supporting Information

S1 Fig [S]-V plot of ATP hydrolysis rate catalyzed by mutated V_1_. The solid lines show fit with the Michaelis-Menten equation.
(TIF)
