# Supplementary figures and images for: The Ingenious Structure of Central Rotor Apparatus in VoV1; Key for Both Complex Disassembly and Energy Coupling between V1 and Vo
Source: PLoS One. 2015 Mar 10;10(3):e0119602. doi: 10.1371/journal.pone.0119602 (PMC4355294; doi:10.1371/journal.pone.0119602)

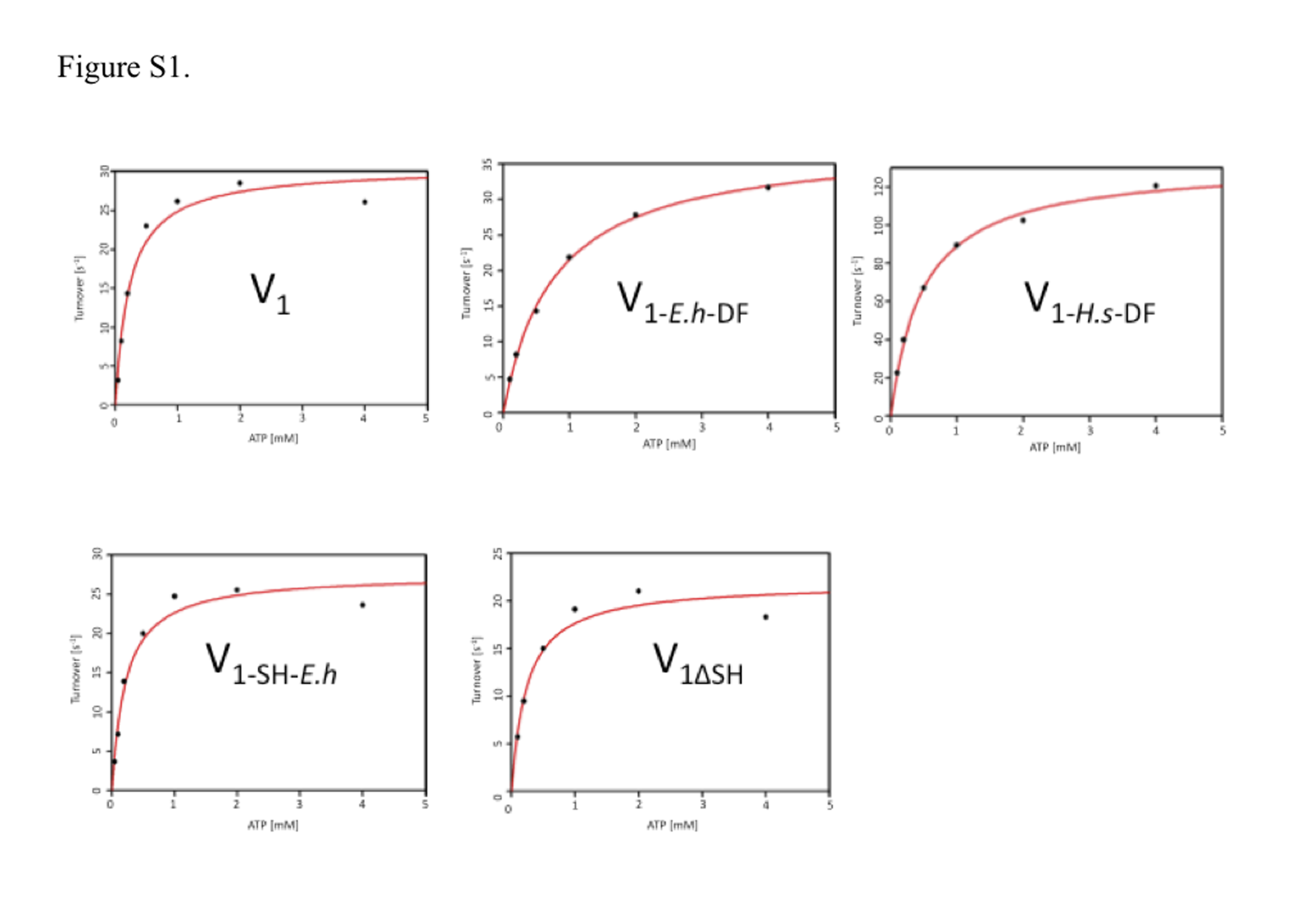

Supplement: S1 Fig — The solid lines show fit with the Michaelis-Menten equation. (TIF) [file pone.0119602.s001.tif]
